# Combination of transjugular intrahepatic portosystemic shunt and antegrade through-the-TIPS coil embolization for bleeding mixed-type ectopic ileal varices

**DOI:** 10.1007/s12328-023-01830-w

**Published:** 2023-07-15

**Authors:** Andrea Michielan, Filippo Vieceli, Cecilia Pravadelli, Luisa Moser, Flora Agugiaro, Giacomo Luppi, Francesco Lorenzo Gatti, Lorenzo Costa, Umberto Maria Rozzanigo

**Affiliations:** 1 Department of Gastroenterology and Digestive Endoscopy Unit, Santa Chiara Hospital, APSS Trento, Trento, Italy; 2 Department of Radiology, Santa Chiara Hospital, APSS Trento, Largo Medaglie D’Oro 9, 38122 Trento, Italy; 3 Department of Diagnostics and Public Health, University of Verona, Verona, Italy

**Keywords:** Ectopic varices, Portal hypertension, TIPS, Embolization, Venous interventions

## Abstract

A 61-year-old man with alcoholic cirrhosis and a history of severe cholecystitis leading to secondary thrombosis of the recanalized paraumbilical vein was admitted to our hospital for recurrent gastrointestinal bleeding and severe anemia. Capsule endoscopy and CT angiography detected profuse bleeding in the proximal ileum from ectopic ileal varices. Hepatic venous-portal gradient (HVPG) measurement was consistent with severe portal hypertension. Persistent bleeding despite transjugular intrahepatic portosystemic shunt (TIPS) placement required a combined approach with antegrade through-the-TIPS coil embolization of the ileal varices.

## Introduction

While esophagogastric varices are a well-known complication of portal hypertension, small bowel, colorectal and extra-intestinal varices represent a rare finding in clinical practice [1]. Among ectopic varices, ileal varices are particularly rare, accounting for only 1.2% of all variceal bleedings [2]. To date, few case reports and case series on management and treatment of bleeding ileal varices have been published, with interventional radiology playing a pivotal role. Treatment of ileal varices was achieved by transjugular intrahepatic portosystemic shunt (TIPS), balloon-occluded retrograde transvenous obliteration (BRTO), percutaneous transhepatic procedure, or antegrade embolization of ileal varices via recanalized paraumbilical veins [3–7]. We report a case of bleeding ectopic ileal varices in a patient with severe portal hypertension and thrombosis of the recanalized paraumbilical vein successfully treated by the combination of TIPS placement and antegrade through-the-TIPS coil embolization.

## Case report

**Fig. 1:**
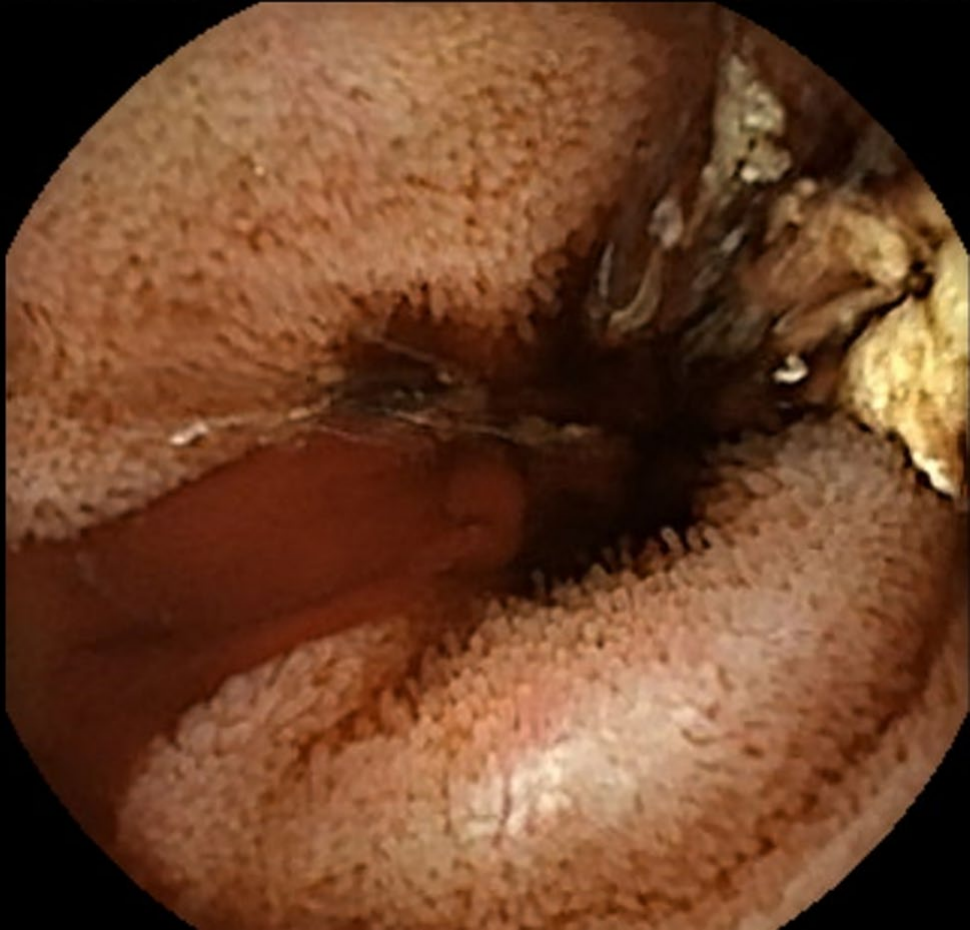
Capsule endoscopy showing active bleeding in the ileum

**Fig. 2:**
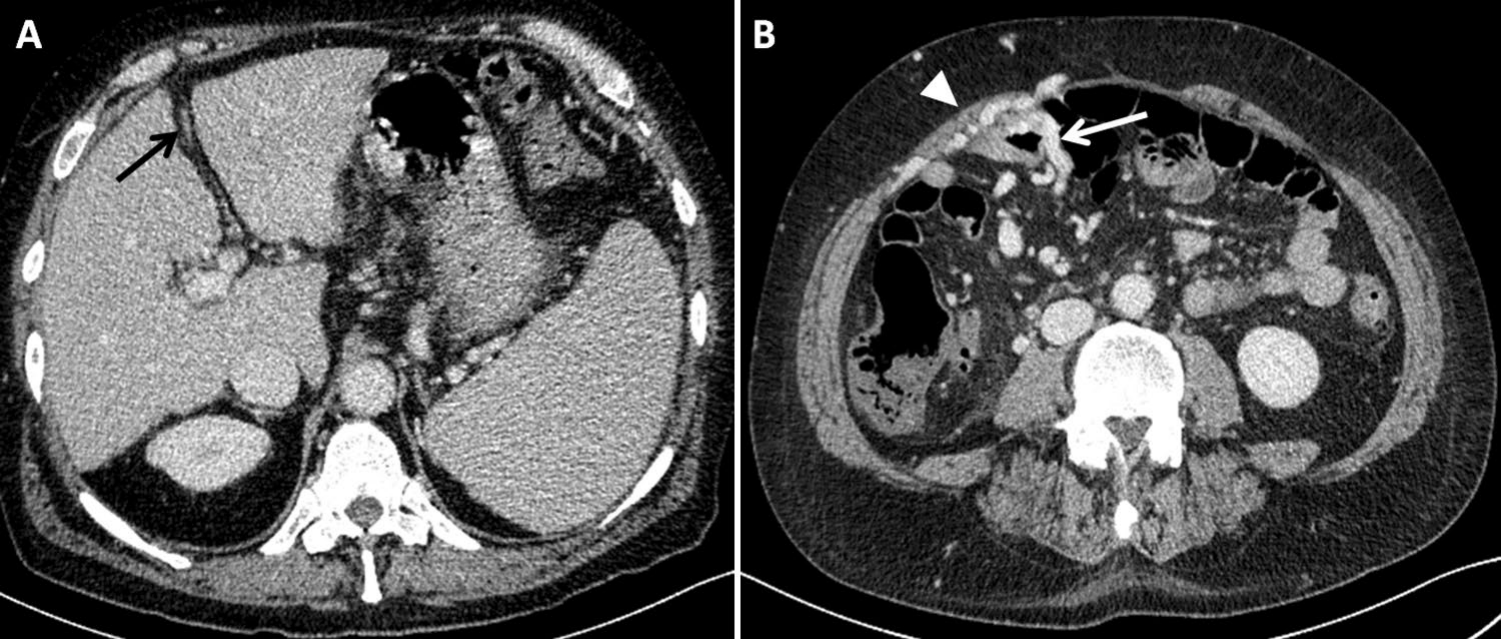
**A**–**B** Venous phase CT scan at the level of the portal vein bifurcation **A** shows cirrhotic liver with the fibrotic remnant of the umbilical vein (black arrow) in the falciform ligament after thrombosis due to cholecystitis; splenomegaly due to portal hypertension is clearly visible. Venous phase CT scan of the paraumbilical region **B** depicts engorged branches of the superior mesenteric vein in connection with submucosal varices protruding into the lumen of the proximal ileum (white arrow); enlarged right epigastric veins (white arrowhead) are also demonstrated in the anterior abdominal wall

A 61-year-old man with alcoholic cirrhosis was admitted to our hospital in December 2022 for melena, hypotension, and severe anemia. He had experienced recurrent gastrointestinal (GI) bleeding for 3 years, despite previous complete endoscopic band ligation of esophageal and ectopic duodenal varices. In 2019, a severe cholecystitis with pericholecystic fluid collections led to secondary thrombosis of the recanalized paraumbilical vein. In November 2022, capsule endoscopy was negative. An emergent esophagogastroduodenoscopy (EGD) revealed no bleeding sources in the upper GI tract, and an ileo-colonoscopy showed dark blood in the distal ileum. Capsule endoscopy was repeated and detected profuse active bleeding in the proximal ileum (Fig. 1), but a subsequent superior mesenteric artery selective angiography was negative for contrast medium extravasation or vascular lesions. CT scan in the venous phase showed ectopic ileal varices in the right lower quadrant in communication with collateral vessels from the superior mesenteric vein and the right inferior epigastric vein (Fig. 2A–B).

The patient presented hypotension (systolic arterial pressure between 70 and 85 mmHg) and severe anemia (hemoglobin 6.1 g/dL) requiring transfusion of more than 20 packs of red blood cells. The clinical course is summarized in Table 1. Other laboratory studies revealed prothrombin time 1.39, platelet count 81,000/µL, bilirubin 1.9 mg/dL and albumin 23 g/L. His Child–Pugh score was B8 and his MELD score was 13. Based on clinical presentation and radiological findings, hepatic venous-portal gradient (HVPG) measurement was performed, confirming a severe portal hypertension (20 mmHg). As the treatment choice, the patient underwent TIPS placement (Gore^®^ Viatorr^®^, 8–10 × 70 mm) with initial balloon dilatation of the stent up to 5 mm. The hepatic encephalopathy score was 0 both before and after TIPS; therefore, ammonia level was not checked. Post-procedure HVPG dropped to 8 mmHg, but intestinal bleeding recurred as well as anemia requiring daily blood transfusions.

**Table 1:**
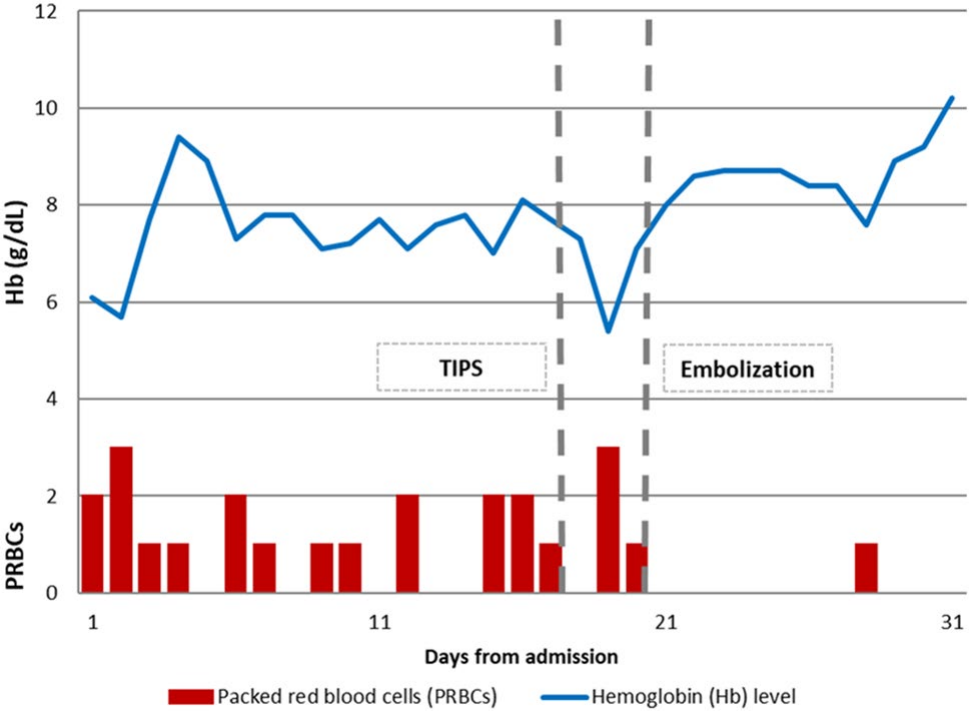
Diagram of the clinical course of the patient after admission highlighting the need of multiple transfusions for severe anemia before definitive treatment with embolization

**Fig. 3:**
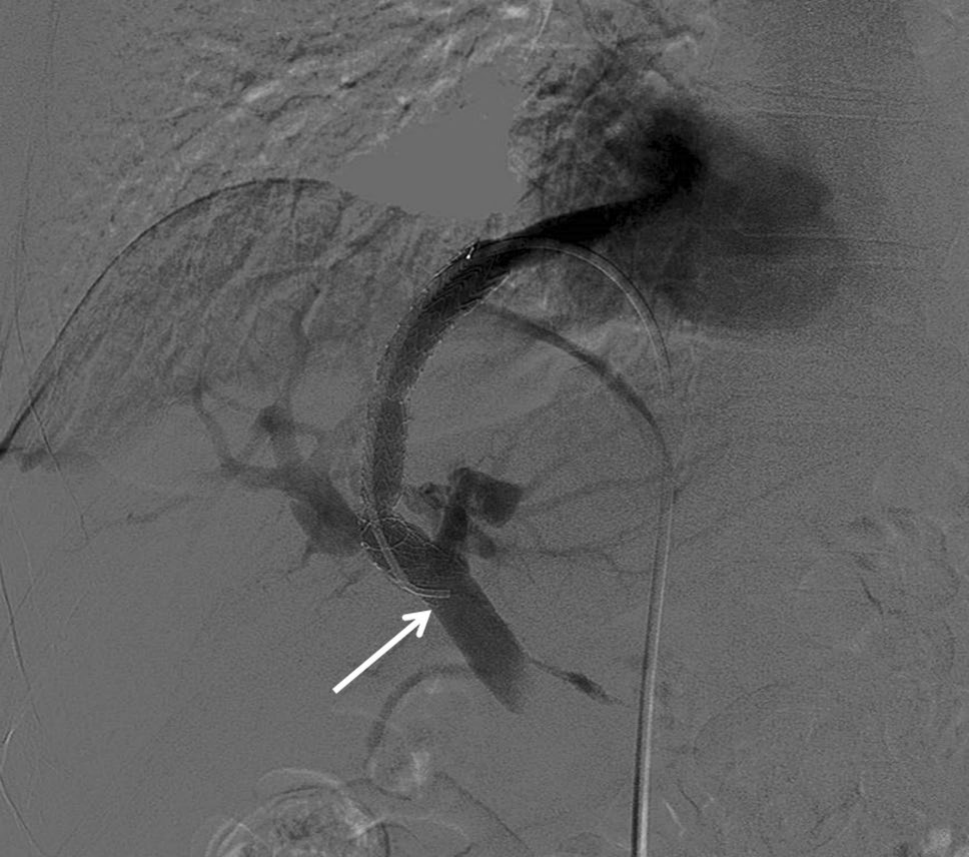
Portography performed with the tip of the guiding catheter (white arrow) positioned through-the-TIPS, after dilatation of the stent up to 6 mm

**Fig. 4:**
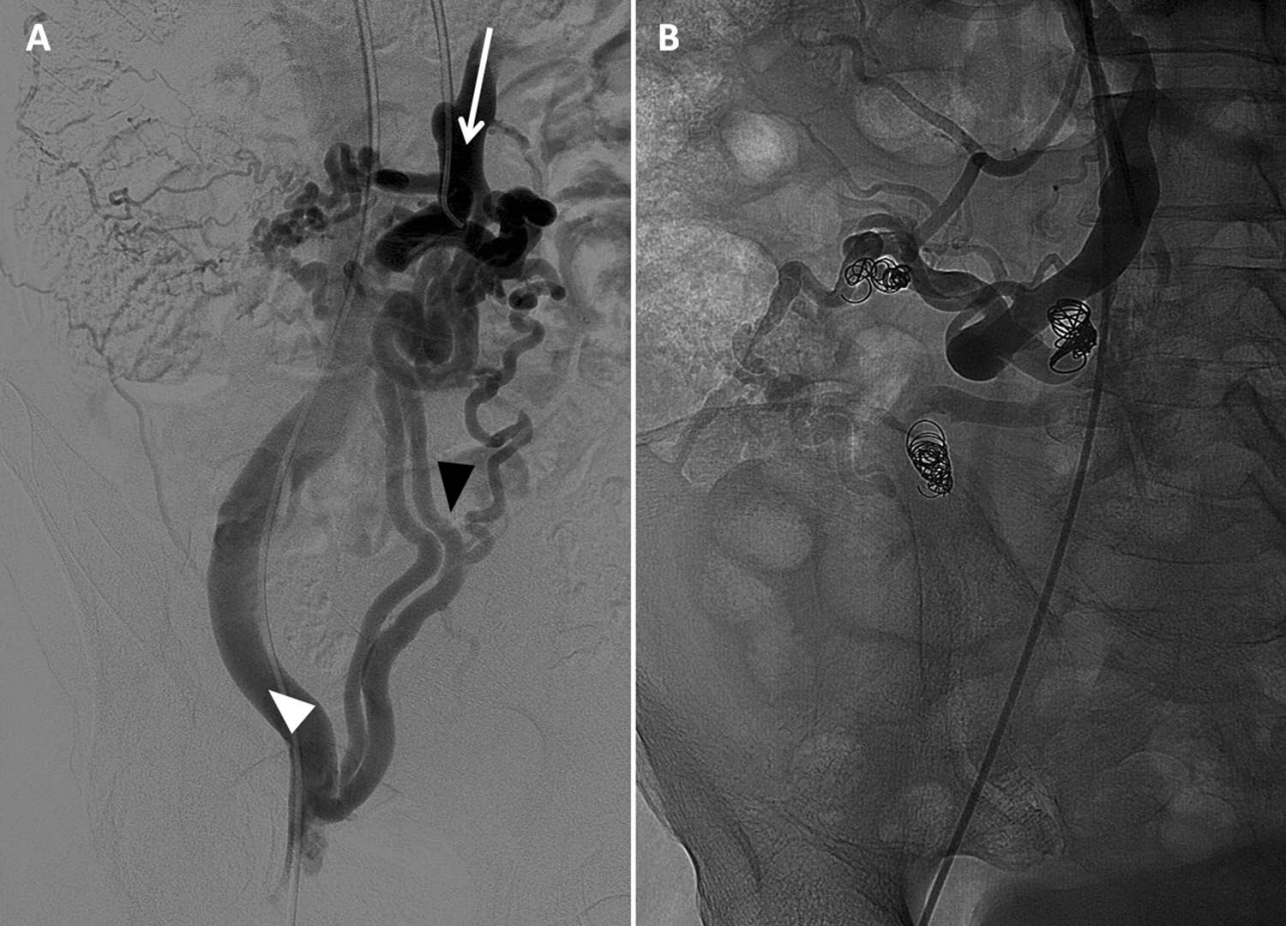
**A**–**B** Phlebography of the superior mesenteric vein (white arrow, indicating also the direction of flow) shows persistent reflux into the ileal varices **(A)**, draining through the inferior epigastric veins (black arrowhead) into the right external iliac vein (white arrowhead). After embolization with multiples coils of the ileal varices **(B),** the injection of contrast medium into the superior mesenteric vein demonstrates absent opacification of the pathological portosystemic shunt through the right inferior epigastric veins

The patient was, thus, scheduled for a concomitant procedure of TIPS dilatation and antegrade through-the-stent ileal varices embolization. Under local anesthesia, a 6-French introducer sheath was placed in the right femoral vein. A 90 cm long guiding catheter (6F Mach 1, Boston Scientific) was inserted through-the-TIPS and dilatation of the stent up to 6 mm was performed (Fig. 3) using a rapid-exchange balloon catheter (6 × 20 mm, 135 cm, Sterling Monorail, Boston Scientific). Access to the mesenteric vein was obtained using a triaxial technique with diagnostic vertebral catheter (4F, 125 cm, Tempo Aqua, Cordis) and a 0.021″ microcatheter (3/2.4F, 150 cm, Renegade STC-18, Boston Scientific). Phlebography showed persistent reflux in the ectopic ileal varices, draining through the inferior epigastric veins into the right external iliac vein (Fig. 4A), despite TIPS dilatation. Therefore, selective coil embolization of the three main ileal varices was performed using both 0.035″ and 0.018″ detachable coils (Interlock Fibered IDC Occlusion System, Boston Scientific) (Fig. 4B). The patient was discharged five days after the procedure without recurrence of bleeding or anemia. Three months later, the latest follow-up visit documented stable hemoglobin (10 g/dL) and improvement of liver function (Child–Pugh score A6). The increase in albumin (39 g/L) was attributable to improved nutritional status after cessation of hospital admissions and prolonged fasting for repeated endoscopic examinations.

## Discussion

### Diagnosis of ectopic varices

Ectopic varices can be divided into non-occlusive or oncotic (Type A) and occlusive (Type B) according to the etiology and are associated with a fourfold higher risk of bleeding compared to esophageal varices [4]. Hemorrhage from ectopic varices is an uncommon clinical finding, which represents only 2–5% of gastrointestinal tract variceal bleeding, mostly occurring in Type A varices due to portal hypertension in liver cirrhosis [1]. Given their rarity and anatomical location, diagnosis and management of bleeding ileal varices is challenging.

Alternative sources of bleeding must be ruled out by imaging in the case of significant GI bleeding in a patient with known portal hypertension, but without classic esophagogastric varices (absent or already treated). Multiphase CT with contrast medium administration may be useful to localize the source of GI hemorrhage in the acute setting. If the clinical suspicion is high, repeated small bowel exploration by capsule endoscopy, preferably during the active bleeding, is warranted.

### Treatment of ectopic varices

Endoscopy, surgery, and interventional radiology have been proposed as alternative treatments depending on the location of the ectopic varices. Given that small bowel can be out of reach for endoscopic therapy and patients with portal hypertension are often unfit for surgery, interventional radiology plays a pivotal role in ileal varices [4]. Furthermore, treatment should include both bleeding control during acute presentation and handling of underlying portal hypertension, to prevent recurrence. TIPS is usually the first choice, thanks to its effectiveness in reducing HVPG and subsequent bleeding control [5]. Nevertheless, TIPS alone was not effective in our case, arguably due to the severity of portal hypertension and the etiology of ectopic varices, which was both Type A and B.

Treatment of ectopic varices using balloon-occluded retrograde transvenous obliteration (BRTO) has been described, but its success rate is less than 50% [4]. Percutaneous transhepatic obliteration (PTO) is known to be effective after failed BRTO, but its application is conditioned by peri-procedural bleeding rates up to 11%, particularly in cirrhotic patients and is not effective in reducing HVPG [4]. The latest international consensus on the management of portal hypertension (Baveno VII) deemed antegrade embolization through-the-stent particularly useful after TIPS, when the portal flow keeps diverting to collaterals despite a drop in HVPG [8]. Some authors even advocate combined TIPS positioning and through-the-stent embolization in the same procedure as the first choice in the case of non-physiological shunts [9].

Antegrade embolization via a recanalized paraumbilical vein has been reported to be effective and safe in managing bleeding ileal varices [6, 7], but our patient was unfit for the procedure because of his previous paraumbilical vein thrombosis. In this case, we considered hazardous the retrograde embolization of the ileal varices through the inferior epigastric veins, because of the risk to increase the variceal congestion by blocking venous drainage into the external iliac vein. We decided to perform a selective coil embolization of the ileal varices using an antegrade through-the-TIPS pathway, according to Liu et al. who performed a balloon-assisted antegrade transvenous obliteration (BAATO) [10], with the aim to stop the high pressure reflux coming from the mesenteric circulation and to preserve the alternative drainage of the ileal loops to the systemic veins.

## Conclusion

We described a rare case of mixed-type ileal varices with severe GI bleeding. Sustained bleeding control was achieved only after through-the-TIPS coil embolization of the varices. This combined approach can be considered in patients with bleeding ectopic varices, particularly in the case of severe portal hypertension.

## References

[1] Broussard KA, Rockey DC (2022). Bleeding ectopic varices: clinical presentation, natural history, and outcomes. J Investig Med.

[2] Watanabe N, Toyonaga A, Kojima S (2010). Current status of ectopic varices in Japan: results of a survey by the Japan Society for Portal Hypertension. Hepatol Res.

[3] Vangeli M, Patch D, Terreni N (2004). Bleeding ectopic varices–treatment with transjugular intrahepatic porto-systemic shunt (TIPS) and embolisation. J Hepatol.

[4] Saad WE, Lippert A, Saad NE (2013). Ectopic varices: anatomical classification, hemodynamic classification, and hemodynamic-based management. Tech Vasc Interv Radiol.

[5] Vidal V, Joly L, Perreault P (2006). Usefulness of transjugular intrahepatic portosystemic shunt in the management of bleeding ectopic varices in cirrhotic patients. Cardiovasc Intervent Radiol.

[6] Fukumoto G, Kimura H, Kanagaki M (2019). Intraabdominal hemorrhage from ruptured ectopic varices treated by antegrade embolization via a recanalized paraumbilical vein. Cardiovasc Intervent Radiol.

[7] Onishi Y, Kimura H, Kanagaki M (2018). Successful embolization of bleeding ileal varices with N-butyl cyanoacrylate via a recanalized paraumbilical vein. Cardiovasc Intervent Radiol.

[8] de Franchis R, Bosch J, Garcia-Tsao G (2022). Baveno VII—renewing consensus in portal hypertension. J Hepatol.

[9] Liu M, Li W, Li P (2020). Ectopic duodenal variceal bleed successfully treated with TIPS and 2 years follow-up: a case report. Radiol Case Rep.

[10] Liu J, Yang C, Huang S (2020). The combination of balloon-assisted antegrade transvenous obliteration and transjugular intrahepatic portosystemic shunt for the management of cardiofundal varices hemorrhage. Eur J Gastroenterol Hepatol.

